# Neurotypical Peers are Less Willing to Interact with Those with Autism based on Thin Slice Judgments

**DOI:** 10.1038/srep40700

**Published:** 2017-02-01

**Authors:** Noah J. Sasson, Daniel J. Faso, Jack Nugent, Sarah Lovell, Daniel P. Kennedy, Ruth B. Grossman

**Affiliations:** 1 School of Behavioral and Brain Sciences, The University of Texas at Dallas, GR41, 800 W Campbell Road, Richardson, TX, 75080-3021, USA; 2 Department of Psychological and Brain Sciences, Indiana University, 1101 E. 10th Street, Bloomington, IN 47405, USA; 3 Department of Communication Sciences and Disorders, Emerson College, 120 Boylston Street, Boston, MA 02116, USA

## Abstract

Individuals with autism spectrum disorder (ASD), including those who otherwise require less support, face severe difficulties in everyday social interactions. Research in this area has primarily focused on identifying the cognitive and neurological differences that contribute to these social impairments, but social interaction by definition involves more than one person and social difficulties may arise not just from people with ASD themselves, but also from the perceptions, judgments, and social decisions made by those around them. Here, across three studies, we find that first impressions of individuals with ASD made from thin slices of real-world social behavior by typically-developing observers are not only far less favorable across a range of trait judgments compared to controls, but also are associated with reduced intentions to pursue social interaction. These patterns are remarkably robust, occur within seconds, do not change with increased exposure, and persist across both child and adult age groups. However, these biases disappear when impressions are based on conversational content lacking audio-visual cues, suggesting that style, not substance, drives negative impressions of ASD. Collectively, these findings advocate for a broader perspective of social difficulties in ASD that considers both the individual’s impairments and the biases of potential social partners.

Individuals with autism spectrum disorder (ASD) are characterized by impairments in social interaction that contribute to broad social disabilities and poor functional outcomes^1^. Across the lifespan, these impairments are associated with smaller social networks and fewer friendships^2^, difficulty securing and retaining employment^3^, high rates of loneliness^4^, and an overall reduced quality of life^2^. Such poor outcomes persist even for individuals with ASD who have average to above average intelligence^5^.

Researchers have long investigated the cognitive and neural differences that contribute to social interaction impairments in ASD^6^. Such work has been invaluable for highlighting behavioral and biological mechanisms of social disability in ASD that may serve as primary targets for treatment. However, social interaction quality is not only predicated upon social ability but also social expression^7^, and many aspects of social presentation are atypical in ASD, including abnormal facial expressivity^8^,^9^,^10^, anomalous use of gaze^11^, lower rates or unusual timing of expressive gestures^12^, violations of personal space^13^, and unusual vocal prosody^14^. These differences in social presentation may affect social interaction quality. Unfamiliar observers judge expressions made by individuals with ASD as more awkward or odd^8^,^15^ and such evaluative judgments could potentially affect interaction quality or even reduce intentions to initiate social interaction altogether. In this way, social interaction impairments in ASD may not only be an individual impairment, but also a relational one^16^ in which the perspectives of others affect the quantity and quality of the social experiences of those with ASD. Indeed, although it has long been established that those with ASD struggle to interpret the mental states of other people^17^, recent findings suggest that neurotypical individuals likewise have difficulty interpreting the mental states of those with ASD^18^,^19^. Thus, difficulties with social interaction for individuals with ASD may be a bidirectional problem, not just an individual one.

Such relational factors are emphasized in the social model of disability^20^,^21^, which posits that those with disabilities may be disadvantaged not only because of their own differences and challenges, but also because of societal structures (e.g., systemic barriers, negative attitudes, and lack of accommodation) that inhibit optimal functioning. However, whether the social disability experienced by those with ASD is affected by the perceptions, behaviors, and social decisions made by their neurotypical peers has largely been overlooked. Are individuals with ASD perceived differently than their neurotypical peers, and if so, do these judgments contribute to the social disability they experience? What aspects of their social presentation influence the formation of judgments made about them and the social behaviors directed towards them?

How people respond to unfamiliar individuals prior to social interaction is governed in large part by first impressions, which are near instantaneous judgments of personality and character traits based upon “thin slices” of information^22^. First impressions are associated with immediate behavioral responses and long-lasting attitudes^23^. Whereas positive first impressions can evoke approach behaviors^24^, negative first impressions often prompt rejection or avoidance behaviors^25^. For individuals with ASD, negative perceptions may relate to the social exclusion they frequently experience^26^ and affect their ability to successfully navigate the social demands necessary for optimal functional outcomes in adulthood^3^.

The present study evaluates first impressions of children and adults with ASD by unfamiliar observers, including same-age peers, and examines their relation to subsequent intentions for social interaction. Unlike prior studies that have assessed attitudes towards autism using survey methods^27^, written vignettes of autism^28^,^29^,^30^, or actors portraying autistic behavior^31^,^32^, we examined impression formation based upon authentic behavior under conditions in which first impressions are truly formed. Here, we present three studies conceived and conducted independently by three research groups assessing observers’ first impressions of—and intentions to socially engage with— children and adults with ASD based upon “thin slices” of their real-world social behavior. These studies aim to assess the ways in which individuals with ASD are initially perceived relative to matched typically developing (TD) controls, examine whether these impressions change with increased exposure, explore the components of social presentation that affect these perceptions, and evaluate whether such perceptions are associated with intentions to socially interact. By exploring these perceptions across different methodologies, stimulus types, and participant cohorts, the studies aim to assess the relative robustness of impressions made by TD individuals towards people with ASD, and determine whether they vary by context, age groups, or methodological variations. Ultimately, this information can highlight previously overlooked social factors that contribute to reduced social interaction quantity and quality for individuals with ASD.

## Study 1

### Method. Participants

Forty adults (20 ASD; 20 TD) served as stimulus participants. The stimulus groups were matched on gender (17 males in each group), and were comparable on both age (ASD mean = 24.5 years, TD mean = 25.0; *p* = 0.786) and full-scale IQ on the WASI (ASD mean = 106.4, TD mean  = 110.5; *p* = 0.293). ASD diagnoses were confirmed by a certified clinician using the Autism Diagnostic Observation Schedule^33^. TD participants reported no ASD diagnosis in themselves or their first-degree family members. 214 undergraduates (164 female; mean age = 21.4) served as rating participants and completed informed consent. Power analysis using GPower 3.1 with an alpha level of 0.05, a power level of 0.8, and an effect of 0.3, which is comparable to related studies with a similar design^8^, determined a sample of 160 participants would be sufficient, but we oversampled relative to this number because previous studies included fewer stimulus participants and fewer presentation modalities. The Institutional Review Board at the University of Texas at Dallas approved this study and all methods were performed in accordance with the relevant guidelines and regulations.

### Stimuli and Materials

Stimuli were created from video recordings of stimulus participants engaging in the “High Risk Social Challenge” task^34^, a 60 s mock audition for a reality/game show. Each video contains bodies and faces of stimulus participants and the first 10 s of social behavior occurring after any content-free introductions (e.g., “Hi TV. This is my audition tape”) was edited into five presentation modalities: (1) audio-only (2) visual-only (3) audio-visual (4) static image and (5) transcript of speech content. Separation of the audio and visual components allows for examining the degree to which these social information sources independently contribute to first impression formation. The static image, taken from the first moment in the video in which the participant is sitting upright with eyes open and not speaking or gesturing, was used to determine whether first impressions of ASD and TD adults differ even in the absence of movement, speech, or conversational content. Finally, the transcript modality aimed to determine whether conversational content, which can differ in ASD and affect social interaction quality^35^, influences impression formation of ASD and TD adults independent of vocal and visual information.

### Procedure

Rating participants provided their judgments individually on a desktop monitor using Qualtrics survey software. To avoid any carry-over effects, rating participants experienced only one presentation modality (e.g., transcript, static frame) for each of the 40 stimulus participants. Each stimulus was rated one at a time on ten items using a four-point scale (0–3). Six items related to traits that are reliably perceived when forming first impressions: attractiveness, awkwardness, intelligence, likeability, trustworthiness, and dominance/submissiveness^14^,^35^. The remaining four items related to behavioral intentions towards the stimulus participant as potential social partners: willingness to live near the stimulus participant, likelihood of hanging out with the stimulus participant in their free time, level of comfort sitting next to the stimulus participant, and likelihood of starting a conversation with the stimulus participant^28^,^30^. Items appeared in a random order between rating participants to account for potential order effects, but were presented in the same order across stimuli for each individual rater. Here, as in Studies 2 and 3, rating participants were not informed of the clinical status of stimulus participants, nor was any reference to autism made anywhere in the task.

## Results

We conducted a 2 (ASD vs. TD stimulus group) by 5 (modality) by 10 (rating) mixed-model ANOVA. A significant main effect of stimulus group (F(1, 38) = 47.33, *p* < 0.001, η^2^ = 0.555) found ASD stimulus participants to be rated less favorably overall (M = 1.46, SD = 0.14) than TD stimulus participants (M = 1.76, SD = 0.15). Significant interactions occurred between: group and modality (F(4, 152) = 9.14, *p* < 0.001, η^2^ = 0.194); group and rating (F(9, 342) = 17.57,  < 0.001, η^2^ = 0.316); and a three-way interaction between group, rating, and modality (F(36, 1368) = 3.10, *p* < 0.001, η^2^ = 0.076). Follow-up pairwise comparisons explored patterns of group differences on each interaction, with Bonferroni correction to control for multiple comparisons and Greenhouse-Geisser correction when Mauchly’s test indicated that the assumption of sphericity had been violated.

**Figure 1:**
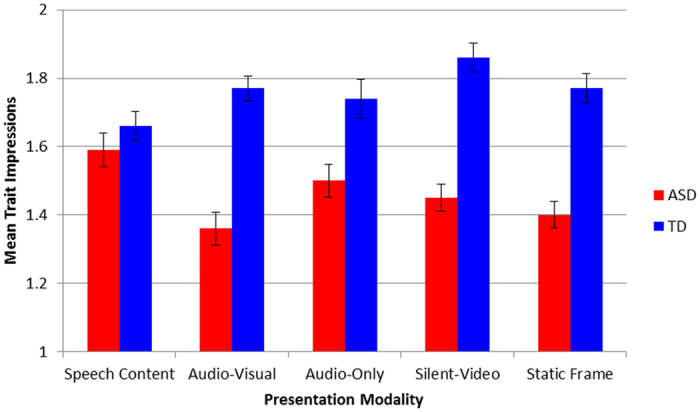
Group effects on each modality. Groups only did not significantly differ on the transcript of Speech Content.

**Figure 2:**
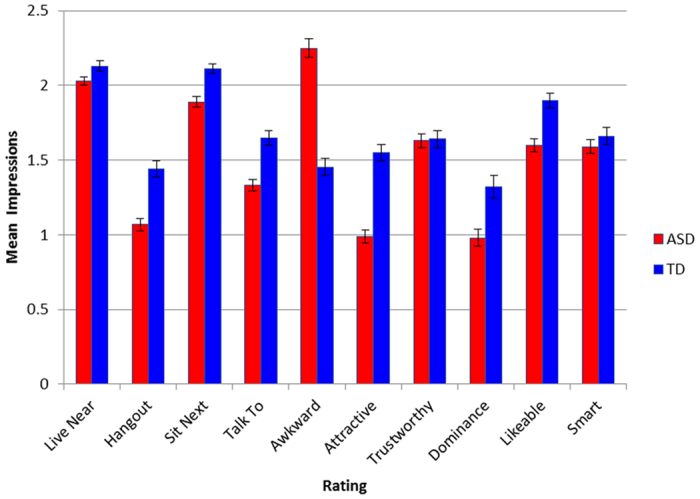
Group effects on each item. ASD group is rated significantly less favorable on every item except Live Near, Trustworthy, and Smart.

For the group by modality interaction (see Fig. 1), ASD stimulus participants were evaluated less favorably than controls across all modalities except for Transcript (t(38) = 1.00, *p* = 0.32, *d* = 0.29). The ASD group was rated significantly less favorably in the audiovisual modality (M = 1.36, SD = 0.16) than in than the Audio (M = 1.50, SD = 0.25, *p* = 0.02, 95% CI [0.01, 0.26], *d* = 0.66) and Transcript (M = 1.59, SD = 0.19, *p* = 0.001, 95% CI [0.08, 0.37], *d* = 1.30) modalities. For the group by rating interaction (see Fig. 2), ASD stimulus participants were rated less favorably than controls on each rating except for trustworthiness t(38) = 0.14, *p* = 0.89, *d* = 0.04), intelligence t(38) = 0.89, *p* = 0.38, *d* = 0.26), and willingness to live nearby t(38) = 2.03, *p* = 0.05, *d* = 0.46), with the largest effect size occurring for the rating of awkwardness (t(38) = 9.37, *p* < 0.001, 95% CI [0.63, 0.98], *d* = 2.96). For the significant three-way interaction between group, rating, and modality, visual modalities (i.e., audio-visual, silent-video, static image) produced less favorable ratings for the ASD stimulus participants than the Audio and Transcript modalities for ratings of awkwardness (F(2.68, 50.85) = 11.78, *p* < 0.001, η^2^ = 0.383), attractiveness (F(2.33, 44.33) = 19.78, *p* < 0.001, η^2^ = 0.510), intent to hangout (F(2.32, 44.15) = 10.16, *p* < 0.001, η^2^ = 0.348) and intent to talk to (F(4, 76) = 6.65, *p* < 0.001, η^2^ = 0.259). In contrast, ratings for the TD group were generally consistent across all modalities, with significant differences only emerging for the silent video modality producing more favorable ratings than the transcript modality for intelligence (F(1.97, 37.41) = 7.43, *p* = 0.002, η^2^ = 0.281), likeability (F(1.76, 33.48) = 5.54, *p* = 0.011, η^2^ = 0.226) and intent to talk to (F(1.91, 36.24) = 4.45, *p* = 0.02, η^2^ = 0.190).

We also explored relationships between individual character and behavioral intent items averaged across modalities (see Table 1). Both groups exhibited strong correlations (all *r*s>.59, *p*s < 0.01) between ratings of trustworthiness, likability, and intelligence and each behavioral intent item. One discrepant pattern between groups did emerge, however: the ASD, but not TD, stimulus participants demonstrated significant negative relationships between awkwardness ratings and the raters’ intent to talk to (r = −0.56, *p* < 0.01) and to hang out with (r = −0.58, *p* < 0.01). Finally, mean ratings across the items did not differ between male and female stimulus participants (*t*(38) = 7.46, *p* = 0.17, Male: *M* = 2.41, *SD* = 0.21; Female: *M* = 2.28, *SD* = 0.20, 95% CI [−0.06, 0.31], *d* = 0.63).

## Study 2

### Method. Participants

A total of 12 adults with ASD (2 female; 10 male) and 16 TD participants (7 female; 9 male) served as stimulus participants. ASD diagnoses were confirmed by the ADOS-2 (Module 4) administered and scored by research reliable personnel. Groups were matched on age (ASD mean = 22.8 ± 3.5; TD mean = 22.8 ± 4.4; t(26) = 0.05, *p* = 0.96) and full-scale IQ (ASD mean = 117.8 ± 11.2; TD mean = 112.0 ± 14.9; t(26) = 1.12, *p* = 0.27). Thirty-seven undergraduate students (18 female, 19 male; mean age = 19.4 ± 1.3) served as raters and provided informed consent. They also received class credit for their participation. The Institutional Review Board of Indiana University approved this study and all methods were performed in accordance with the relevant guidelines and regulations.

### Stimuli and Materials

Stimulus participants sat directly across from an experimenter while responding to a brief set of open-ended casual questions (e.g., “Have you seen any good movies recently?”). The experimenter was instructed to respond appropriately and attempt to engage the participant in a natural dialogue. All participants were familiar with the experimenter from having participated in previous studies in the laboratory. The experimenter recorded video using wearable point-of-view video recording glasses (Pivothead HD; recorded in 1080p), thus providing a direct first-person point of view of the social interaction. All but five participants also completed the UCLA Loneliness Scale.

We used MATLAB (v2014b; MathWorks, Natick, MA) to randomly select 10 still frames (henceforth, images) from each video to be used as stimuli. Images were only excluded if the subject was not near the middle of the frame or if the image was blurry (both due to head movement of the experimenter), or if the stimulus participant’s eyes were closed. These images were then cropped to eliminate some of the background and show the person from approximately their torso and above.

### Procedure

Raters were instructed that they would be viewing a variety of photographs of individuals taken during a typical social interaction, and their task was to make various judgments based on these images. Each rater completed 3 experimental blocks, with each block corresponding to a different rating question: (1) “How socially awkward is this person?” (2) “How approachable is this person?” (3) “Would I see myself being friends with person?” Stimuli within each block were randomized, and each stimulus participant was seen a total of 10 times per block. Male raters only rated male stimulus participants (190 stimuli per block), and female raters only rated female stimulus participants (90 stimuli per block).

Ratings were made using a non-graduated slider, with ends labeled and representing the extremes of the judgment (e.g., “not socially awkward” vs. “very socially awkward”). These extremes were quantified as ‘0’ and ‘1’, with intermediate values falling in between this range corresponding to the location of the sliding bar. Participant responses were stored with a mouse click, and the next stimulus was shown after a 1 second inter-trial interval. There were no time constraints for responding. The experiment was presented using MATLAB and Psychtoolbox (PTB3)^37^.

## Results

**Figure 3:**
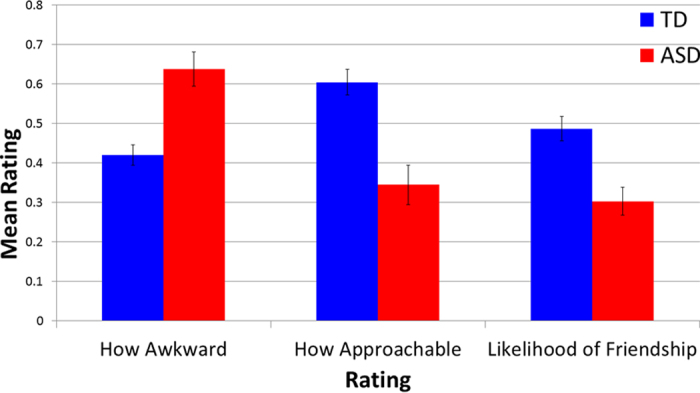
Individuals with ASD (red) are immediately judged more negatively than TD controls (blue) in each of the three rating categories (all *ps* < 0.001).

To examine initial first-impression judgments, we first limited our analysis to the rating on the first image from each stimulus participant. A 3 (rating categories) x 2 (group) repeated measures ANOVA revealed a main effect of rating category [F(2, 25) = 10.517, *p* < 0.001; η^2^ = 0.457] and group [F(1, 26) = 23.348, *p* < 0.001, η^2^ = 0.473], but no group x rating category interaction [F(2, 25) = 1.932, *p* = 0.166, η^2^ = 0.134]. [Note that scores for the awkwardness were reverse scored so that higher scores reflect positive judgments for all questions.] Across all three rating categories, individuals with ASD were rated less favorably than TD controls (Awkwardness: t(26) = 4.51, *p* < 0.001, 95% CI [0.12, 0.32], *d* = 1.68); Approachability: t(26) = 4.57, *p* < 0.001, CI [−0.38, −0.14], *d* = 1.71; Likelihood of being friends: t(26) = 3.91, *p* < 0.001, 95% CI [−0.28, −0.09], *d* = 1.50) (see Fig. 3). Ratings of individuals were highly consistent across the three judgment tasks (ASD pairwise correlations were all r > 0.95, p < 0.001; TD control pairwise correlations were all r > 0.69, p < 0.003). While the small sample of female ASD participants precludes formal statistical testing, our results provide preliminary support for the claim that these effects are at least as strong (if not stronger) for women with ASD (ASD men vs. ASD women: awkwardness: 0.61 vs. 0.80; approachability: 0.38 vs. 0.19; likelihood of being friends: 0.33 vs. 0.19).

**Figure 4:**
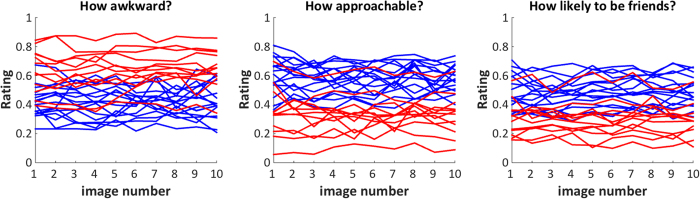
Ratings are very stable over repeated exposure to the same person. Each line represents the mean rating provided for one stimulus participant across image exposure number (blue = TD; red = ASD). Furthermore, note that ratings of stimulus participants are highly consistent across these three judgments tasks (i.e., individuals who scored high on awkwardness scored low on approachability and friendship, and vice versa). For example, the one ASD participant with very typical ratings for awkwardness is the same participant that is rated high on the approachability and friendship ratings.

We next asked whether ratings of an individual by others were stable across repeated exposure, or whether they changed over time. Each rater saw each stimulus participant 10 times over the course of each block. For each rating category and each rater, we fit a line across their 10 ratings of each stimulus participant. Then, we averaged the slopes of the lines across raters for each stimulus participant, and carried out a 3 (rating category) x 2 (group) repeated measures ANOVA as above, but with slope as the dependent variable. There was no effect of rating category [F(2, 25) = 0.79, *p* = 0.466, η^2^ = 0.06], group [F(1, 26) = 0.24, *p* = 0.63, η^2^ = 0.01], or group x rating category interaction [F(2, 25) = 2.40, *p* = 0.111, η^2^ = 0.16]. Furthermore, for all ASD and TD stimulus participants, slopes were not different from zero (6 one-sample t-tests), demonstrating that ratings were reliable over repeated exposure [all *p*s > 0.11] (see Fig. 4). Finally, individuals with ASD reported greater levels of loneliness than TD control participants [ASD mean = 54.5 (11.5); Control mean = 40.2 (8.6); t(21) = 3.37, *p* = 0.0029; *d* = 1.41].

## Study 3

### Method. Participants

14 boys, seven with ASD and seven TD controls, served as stimulus participants. The groups did not differ significantly on age (ASD mean = 12:1 [134.14 months], TD mean = 12:4 [147.43 months]; *p* = >0.250), IQ on the Leiter^38^ (ASD mean = 105.14, TD mean = 114.57; *p* = 0.200), or receptive vocabulary on the PPVT^39^ (ASD mean = 119.14, TD mean = 131.71; *p* = 0.189). ASD diagnosis was confirmed by trained clinicians using the ADOS^32^, and all TD participants scored below the ASD threshold on the CARS^40^. 125 adults and 33 adolescents served as rating participants. Data from adult raters were collected via the online crowdsourcing platform Amazon Mechanical Turk (MTurk). Because facial expressions and prosody are highly dependent on cultural-linguistic context, we excluded participants who did not grow up and live in the US or spoke American English as their primary language, or who did not complete the task. The final sample of adult raters (n = 98) had an average age of 31:4 (range = 19–64), and a gender distribution of 53 male, 44 female, and one not specified. 33 TD adolescents (23 male, 10 female) with a mean age of 13:1 (range = 10–16:11) provided ratings in the laboratory. Data from this sample was analyzed separately to examine first impressions from raters more similar in age to the stimulus participants. The Institutional Review Board of Emerson College approved this study and all methods were performed in accordance with the relevant guidelines and regulations.

### Stimuli and Materials

Stimulus participants were recorded during a task in which they were asked to retell brief stories containing happiness; surprise, fear, and anger (see ref. 9). Brief video clips (2–4 seconds) were extracted representing all four emotions and containing one complete sentence or phrase each. Video clips were selected based on video/audio quality, grammatical accuracy of the sentence productions, and were matched by expressed emotion across the two diagnostic groups. A maximum of two videos per stimulus participant were used with some children appearing in only one video due to availability of stimuli with acceptable video and audio quality, resulting in a total of 24 videos with 12 videos from each of the two subgroups. We created two pseudorandomized sequences, which were counterbalanced across participants.

### Procedure

Adult rater participants completed the tasks remotely via M-Turk and electronically provided informed consent. All task directions were displayed before each video presentation. For each trial, participants viewed one of the 24 video clips and answered five questions using a non-graduated slider bar with “not likely” and “very likely” as the two anchor points. We chose a non-graduated slider to allow maximum freedom of judgment for participants, rather than forcing more categorical choices on a numeric scale. Participants were asked to rate the children’s overall awkwardness, as well as their likelihood to start a conversation with others, have a lot of friends, get along well with others, and spend a lot of time by themselves. Average slider response was calculated for each prompt/question within each diagnostic group, using a range of 0–100 with 50 being the neutral or mid-point on the slider bar. Adolescent rater participants completed a similar task via a computer within the laboratory, but only rated three items provided by the adult raters: awkwardness, getting along well with others, and spending a lot of time by themselves.

## Results

**Figure 5:**
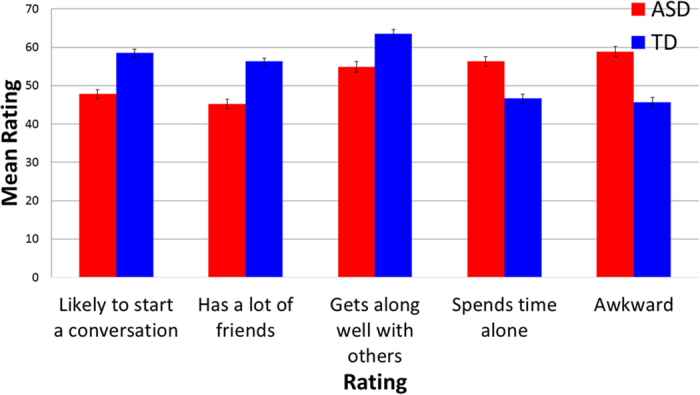
Ratings for ASD stimulus participants (red) are all significantly less favorable than for TD controls (blue). All *ps* < 0.001.

Scores for two items (“awkwardness” and “spending time by themselves”) were reversed scored in all analyses so that higher ratings reflect more positive judgments for all items. Data from adult raters were analyzed first. We conducted a 2 (stimulus producer group: ASD vs TD) by 5 (question) repeated measures ANOVA, which revealed a main effect for stimulus participant group (*F*(1, 97) = 104.99, *p* < 0.001, η^2^ = 0.52) and a main effect for question (*F*(4, 388) = 46.32, *p* < 0.001, η^2^ = 0.32, as well as a significant interaction between question and stimulus participant group *F*(4, 388) = 4.98, *p* = 0.001, η^2^ = 0.05). Ratings can be found in Fig. 5.

We conducted follow-up pairwise t-tests to compare item responses between groups, using Bonferroni adjusted alpha levels of.01 per test to adjust for multiple comparisons. Results show that children with ASD were rated as significantly less likely to start a conversation with others (*t*(97) = −7.59, *p* < 0.001, 95% CI [−13.47, −7.88], *d* = 0.95), less likely to have a lot of friends (*t*(97) = 9.85, *p* < 0.001, 95% CI [−13.29, −8.83], *d* = 1.06), less likely to get along well with others (*t*(97) = 7.76, *p* < 0.001, 95% CI [−10.85, −6.43], *d* = 0.69), more likely to spend time by themselves (*t*(97) = 7.65, *p* < 001, 95% CI [−12.30, −7.23], *d* = 0.80), and were judged to be significantly more awkward than the TD controls in the videos (*t*(97) = 10.05, *p* < 0.001, 95% CI [−15.93, −10.68], *d* = 1.03). Lower awkwardness ratings significantly correlated with greater perceived likelihood of starting a conversation with others (*r* = 0.517, *p* < 0.001), having a lot of friends (*r* = 0.573, *p* < 0.001), getting along well with others (*r* = 0.399, *p* < 0.001), and with the perceived likelihood of spending a lot of time alone (*r* = 0.780, *p* < 0.001). These relationships remain significant when examining stimulus participant groups separately: likelihood of starting a conversation with others (ASD: *r* = 0.650, *p* < 0.001; TD: *r* = 0.340, *p* = 0.001), having a lot of friends (ASD: *r* = 0.678, *p* < 0.001; TD: *r* = 0.555, *p* < 0.001), getting along well with others (ASD: *r* = 0.509, *p* < 0.001; TD: *r* = 0.357, *p* < 0.001), and perceived likelihood of spending a lot of time alone (ASD: *r* = 0.819, *p* < 0.001; TD: *r* = 0.685, *p* < 0.001).

To examine whether patterns held when raters were similar in age to the stimulus participants, a 2 (stimulus participant group: ASD vs TD) by 3 (question) repeated measures ANOVA was conducted using a sample of adolescent raters. We found a main effect for stimulus participant group (*F*(1, 32) = 15.97, *p* < 0.001, η^2^ = 0.333), but no main effect of question (*F*(2, 64) = 0.12, *p* = 0.887, η^2^ = 0.004), and no question by stimulus participant group interaction (*F*(2, 64) = 1.04, *p* < 0.36, η^2^ = 0.032). Follow-up pairwise t-tests using Bonferroni adjusted alpha levels of.0167 per test compared responses to each question between stimulus participant groups and show that children with ASD were rated by adolescents as significantly less likely to get along well with others (*t*(32) = 4.69, *p* < 0.001, 95% CI [−38.8, −15.31], *d* = 0.39) and more likely to spend time by themselves (*t*(32) = 3.75, *p* = 0.001, 95% CI [11.88, 40.11], *d* = 0.32). Awkwardness, however, did not reach significance (*t*(32) = 1.94, *p* = 0.06, 95% CI [−0.85, 35.19], *d* = 0.25).

## Discussion

Across three independent studies using distinct samples and a variety of methodological approaches, observers’ first impressions of individuals with ASD engaging in real-world social behavior were found to be robustly less favorable than those of matched TD controls. These negative first impressions were consistent for both adults and children with ASD, for static as well as dynamic stimuli, for both brief (2–4 s) and longer (10 s) glimpses of social behavior, and did not change with repeated exposure. Further, because these impressions were associated with reduced intentions to socially engage by observers, they may reflect a previously under-recognized contributor to the reduced quantity and quality of social interaction experienced by individuals with ASD.

Our findings show that negative first impressions of adults with ASD occurred only when audio and/or visual information was present, and not when the transcript of their speech content was evaluated (Study 1). This discrepancy suggests that social presentation style rather than the substantive content of social speech drove negative impression formation of individuals with ASD. Supporting this conclusion, a static image was sufficient for generating negative first impressions of those with ASD and including additional information, such as body movement or voice, did not worsen them further. In contrast, first impressions of TD controls improved with the addition of a visual information, suggesting that unlike the ASD group, visual cues helped rather than hurt the impressions they made on observers. We also determined that negative impressions extended beyond perceived social competence to judgments of likeability (reduced), attractiveness (reduced), and submissiveness (increased) (Study 1). However, negative impressions did not occur for all evaluated traits, with the two groups not differing on ratings of perceived intelligence or trustworthiness. The lack of group differences on these traits suggest that the social presentation differences in the ASD group may lead to more negative evaluations of traits associated with social appeal and approach behaviors (e.g., awkwardness, attractiveness, likability), than those associated with competence (intelligence) and character (trustworthiness).

We also demonstrate that negative impressions remain stable across multiple thin-slice judgments. A series of randomly selected static images of college-aged individuals with ASD collected from a first-person perspective during social interaction were consistently rated as less approachable and more awkward than matched controls, with observers indicating a lower likelihood of being friends with members of the ASD group (Study 2). These ratings were stable across ten exposures of different randomly-selected images of each individual, suggesting that the negative impressions of the ASD group were not driven by poorer or non-representative image capture or by sequence effects, but rather by reliable differences in social presentation in ASD leading to consistent negative evaluation. This finding of negative evaluations of individuals with ASD is also emphasized by the consistency of results across all three studies, which include static and dynamic stimuli, as well as representations of different age groups.

Adult and school-aged observers also report negative perceptions of adolescents with ASD (Study 3), indicating that negative first impressions persist even when observers are similar in age to those being evaluated. The extension to school-aged children is important given that individuals with ASD generally receive the greatest amount of social skills intervention during this developmental period^41^, which decreases precipitously after high school^42^. Thus, despite being at an age when intense attention is often paid to social skills training, the ASD children in Study 3 are nonetheless rated poorly by both adults and same-age observers. Negative first impressions by adults may affect how children with ASD are perceived and treated by educators, and similar impressions by same-age observers may limit the formation of social networks and friendships. The establishment of a peer social network during early adolescence facilitates identity formation, access to resources, stress and coping support, and general well-being^43^, and the findings here may reflect another obstacle impeding the establishment of such social connections for youth with ASD above and beyond their already existing difficulties with social skills and social understanding.

Findings across the three independent studies were remarkably consistent despite using distinct samples and methods. Taken together, they offer strong evidence that the social presentations of individuals with ASD, particularly their non-verbal cues, including prosody, facial expressions, and body posture, are perceived less favorably and are associated with reluctance on the part of observers to pursue social engagement. This is particularly important given that individuals with ASD self-reported much greater feelings of loneliness than controls (Study 2). This is consistent with prior research indicating that individuals with ASD do not differ from their TD peers in their desire for relationships^4^, and suggests an unmet need for social experiences in ASD. Negative first impressions may serve as a barrier to fulfilling this desire for social interaction, as approach and withdrawal behavior towards novel social partners is based on subjective perceptions regardless of their accuracy^44^. In turn, this may limit opportunities in ASD for developing social connections and friendships, as well as the intergroup contact necessary for mitigating negative biases when present^45^. We explored the effect of repeated exposure to images of the same individual and found no changes in perceptual ratings (Study 2). However, repeated exposure to images is not equivalent to increased familiarity, and thus the current study cannot assess whether increased personal contact with individuals with ASD reduces negative impressions over time. Future studies are encouraged to explore this possibility, as such evidence would be consistent with a large literature in social psychology indicating that increasing intergroup contact reduces bias and prejudice^45^. In order to determine the effect of increased familiarity on these types of ratings, future studies would need to pursue a longitudinal approach, collecting interpersonal ratings at several time points over the course of weeks or months.

It is also important to explore specific components of visual and/or auditory presentation that may lead to negative impressions of young adults with ASD (e.g., body posture, prosody, grooming, and fashion). Several of the stimuli used across the three studies were videos and there is some evidence in the literature that the movement patterns of individuals with ASD are atypical and may represent a salient cue of awkwardness or difference to TD observers^46^,^47^. Kinematic analyses of facial expressions in this population point to subtle dynamic differences related to the complexity of dynamic transitions^45^ as well as symmetry of movement patterns between face regions^48^, which could represent at least some of the cues potential conversation partners use to form their first impressions of individuals with ASD. However, negative judgments of people with ASD in our studies were not limited to video stimuli, but remained equally robust for static images, indicating that the rougher movement patterns of individuals with ASD^46^,^48^ are not solely to blame for this phenomenon. It remains an open question whether videos would lead to more negative first impressions than static images within more socially demanding contexts such as the dynamic interaction in Study 2 that required spontaneous responses to questioning, rather than the one-sided social presentations elicited in studies 1 and 3.

Recent studies also suggest the presence of autism-specific dysmorphology, specifically related to distances between facial features^48^ that could lead to perception of atypicality even when looking only at static images. However, significant dysmorphic features most commonly characterize individuals with more significant autism symptoms and severity^48^. This does not describe the individuals with preserved language and cognitive skills who appeared in the stimuli for the studies presented here and therefore cannot explain the rapid and robust negative evaluations reported here. Additionally, individuals with ASD are rated less favorably than their TD peers even in audio-only conditions (Study 1), indicating a significant contribution of prosodic features to the negative first impressions we report^14^. Based on evidence in the literature and the data presented here, we propose that negative first impressions of ASD are not founded on any one feature of expression, but rather represent an effect of subtle physical, dynamic, and auditory cues of presentation that can also include additional features, such as clothing choices, grooming habits, gaze patterns, or body posture.

The consistency of findings across these studies using different methodologies and stimulus types indicates that the foundation of these negative perceptions is complex and difficult to isolate. If our goal is to improve social interactions for individuals with ASD, it may therefore be equally important to educate others to be more aware and accepting of social presentation differences, rather than trying to change the many interwoven factors of self-presentation that mark the expressions of individuals with ASD as atypical. Given the social cognitive difficulties in perspective taking associated with autism^6^, some individuals with ASD may lack insight about how their social presentation is viewed by potential social partners. Others, however, may be more cognizant of these perceptions but are comfortable in their self-expression. For them, intervention strategies targeting awareness and acceptance among TD peers in their social environments may be a more sensitive and accommodating approach than encouraging impression management strategies.

The studies reported here should be viewed in the context of several limitations. Although the real-world social presentations used in each study provided a more naturalistic portrayal of actual social behavior in ASD compared to previous work using actors or vignettes, they may not fully reflect how impression formation occurs during live social interaction. We only included explicit judgments made of those with and without ASD. In addition to what participants indicate explicitly, their biases may also play out in more implicit ways. Future studies may seek to address whether implicit biases towards those with ASD parallel the more explicit findings reported here. Further, all participants with ASD in this study were intellectually-capable, and findings may not generalize to cognitively impaired populations. Additionally, while ratings did not differ for male and female stimulus participants in studies 1 and 2, sample sizes of females were small and future studies should examine whether patterns differ by gender. Finally, these studies present only group-wise comparisons and do not address individual differences among those with ASD, nor whether individual characteristics of the raters (e.g., gender, personality, etc.) affect the results reported here.

These limitations notwithstanding, the current project provides convergent evidence across three independent studies that first impressions of individuals with ASD are significantly less favorable than those of matched TD controls, and are associated with greater reluctance on the part of observers to pursue social engagement. The data demonstrate that negative impressions are formed across age groups and based on a range of features, including visual, auditory, dynamic, and static cues, indicating that these impressions are not context- or stimulus-dependent and are likely to persist across a range of real-world social situations. Taken together, these findings suggest that social interaction difficulties in ASD are not solely an individual impairment but also a relational one, and consideration of both of these factors is necessary for a full understanding of social impairment in ASD. The reluctance of TD individuals to engage in social interactions with their ASD peers further limits the opportunities for individuals with ASD to practice their already fragile social skills. This can have a significant negative impact on the ability of socially aware and socially interested individuals with ASD to improve their social communication abilities and work toward more successful social integration. Therefore, intervention and education approaches that target both those with ASD as well as their TD peers may offer a more comprehensive approach for improving social and functional outcomes in autism.

## Additional Information

**How to cite this article**: Sasson, N. J. *et al*. Neurotypical Peers are Less Willing to Interact with Those with Autism based on Thin Slice Judgments. *Sci. Rep.* **6**, 40700; doi: 10.1038/srep40700 (2016).

**Publisher's note:** Springer Nature remains neutral with regard to jurisdictional claims in published maps and institutional affiliations.

## Figures and Tables

**Table 1:** Zero-order Correlations for ratings of traits and behavioral intent items.

Variable	1	2	3	4	5	6	7	8	9	10
1. Live Near	—	0.78**	0.94**	0.83**	−0.16	0.55*	0.94**	−0.54*	0.86**	0.70**
2. Hangout	0.87**	—	0.88**	0.98**	−0.58**	0.88**	0.72**	−0.09	0.88**	0.59**
3. Sit Next	0.95**	0.94**	—	0.94**	−0.38	0.72**	0.88**	−0.35	0.90**	0.71**
4. Talk To	0.88**	0.96**	0.94**	—	−0.56**	0.85**	0.78**	−0.15	0.92**	0.63**
5. Awkward	0.24	−0.08	0.11	−0.17	—	−0.59**	−0.19	−0.60**	−0.53*	−0.21
6. Attractive	0.42	0.72**	0.53*	0.63**	−0.32	—	0.41	0.12	0.64**	0.56**
7. Trust	0.94**	0.85**	0.92**	0.89**	0.08	0.35	—	−0.57**	0.85**	0.58**
8. Dominance	−0.88**	−0.68**	−0.84**	−0.67**	−0.57**	−0.20	−0.79**	—	−0.25	−0.08
9. Likeable	0.81**	0.92**	0.89**	0.94**	−0.23	0.61**	0.88**	−0.58**	—	0.53*
10. Smart	0.91**	0.76**	0.86**	0.78**	−0.22	0.27	0.90**	−0.82**	0.71**	—

Note: Values below the diagonal (blue) reflect correlations within TD stimulus participants. Values above the diagonal (red) reflect correlations within ASD stimulus participants. ^*^p < 0.05. ^**^p < 0.01.
